# Effects of Neural Morphology and Input Distribution on Synaptic Processing by Global and Focal NMDA-Spikes

**DOI:** 10.1371/journal.pone.0140254

**Published:** 2015-10-13

**Authors:** Alon Poleg-Polsky

**Affiliations:** Synaptic Physiology Section, National Institute of Neurological Disorder and Stroke, National Institutes of Health, Bethesda, Maryland, United States of America; University of Connecticut, UNITED STATES

## Abstract

Cortical neurons can respond to glutamatergic stimulation with regenerative N-Methyl-D-aspartic acid (NMDA)-spikes. NMDA-spikes were initially thought to depend on clustered synaptic activation. Recent work had shown however a new variety of a global NMDA-spike, which can be generated by randomly distributed inputs. Very little is known about the factors that influence the generation of these global NMDA-spikes, as well the potentially distinct rules of synaptic integration and the computational significance conferred by the two types of NMDA-spikes. Here I show that the input resistance (R_IN_) plays a major role in influencing spike initiation; while the classical, focal NMDA-spike depended upon the local (dendritic) R_IN_, the threshold of global NMDA-spike generation was set by the somatic R_IN_. As cellular morphology can exert a large influence on R_IN_, morphologically distinct neuron types can have dissimilar rules for NMDA-spikes generation. For example, cortical neurons in superficial layers were found to be generally prone to global NMDA-spike generation. In contrast, electric properties of cortical layer 5b cells clearly favor focal NMDA-spikes. These differences can translate into diverse synaptic integration rules for the different classes of cortical cells; simulated superficial layers neurons were found to exhibit strong synaptic interactions between different dendritic branches, giving rise to a single integrative compartment mediated by the global NMDA-spike. In these cells, efficiency of postsynaptic activation was relatively little dependent on synaptic distribution. By contrast, layer 5b neurons were capable of true multi-unit computation involving independent integrative compartments formed by clustered synaptic input which could trigger focal NMDA-spikes. In a sharp contrast to superficial layers neurons, randomly distributed synaptic inputs were not very effective in driving firing the layer 5b cells, indicating a possibility for different computation performed by these important cortical neurons.

## Introduction

Regenerative dendritic spikes are ubiquitously found in many neural types and can have a profound effect on the rules of synaptic integration and play a predominant role in cellular computations. For example, the apical dendrites of layer 2/3 and 5 cells, which integrate synaptic input over the whole apical tuft region, effectively forming a compartment of input summation that is relatively independent from the rest of the cell[1–4]. More recently, dendritic spikes were shown to be generated directly by synaptic inputs, in the form of focal NMDA-spikes [5–12]. The integrative region of a single focal NMDA-spike was shown to be restricted to 70–100 μm, which is typically less than the length of an individual dendrite. Inputs outside of this integration zone tend to summate independently [6]. This separation of input integration can in principle allow multi-compartmental integration of synaptic inputs [6, 13], where dendrites preprocess the synaptic information independently of each other and the cell body[14–17].

Surprisingly, an NMDA dependent spiking mechanism was recently described for a completely different synaptic activation pattern. Random stimulation of presynaptic fibers, which most certainly did not lead to activation of synaptic clusters, repeatedly generated NMDAR dependent spikes in cortical layer 4 spiny stellate neurons [18]. Simulations faithfully recreated experimental results, showing that sometimes NMDA spiking mechanism can link synapses located hundreds of microns away. This raises the possibility of an NMDA-spike dependent integration compartment that encompasses the whole dendritic tree of a cell. In this scenario many dendrites participate in a global form of an NMDA-spike that doesn’t require synaptic clustering or in fact any rule of synaptic placement [18].

This finding raised many questions regarding the properties of the NMDA-spikes and synaptic integration schemes they may enable. Are these two distinct types of NMDA-spikes, or do they represent a continuum? What factors determine whether a focal or a global form of NDMA spike will be generated by a synaptic input? Do different cells or dendritic trees have similar rules of NMDA-spike initiation? If so, is synaptic integration influenced in a predictable manner between various cell classes?

In this work I employed computer simulations to address these questions. First I investigated the electrical and morphological factors that may influence the NMDA-spiking mechanism in a simplified neuronal morphology. These simulations revealed that the dendritic and the somatic input resistances set the stage for spike generation parameters. Thus complex effects of elaborate neuronal morphologies on NMDA-spike initiation can be collapsed into just two parameters. When applied to synaptic integration in realistic cortical morphologies, the simulations revealed a distinct generation properties of the focal and global NMDA-spikes and integration compartments between deep and superficial cortical layers; while deep cortical neurons had a sharp distinction between focal and global NMDA-spikes and exhibited multicompartmental between-branch processing, superficial layers neurons, with their smaller somas and dendritic trees, were prone to global NMDA-spike generation, where the entire cell was a single integration compartment.

## Results

**Fig 1:**
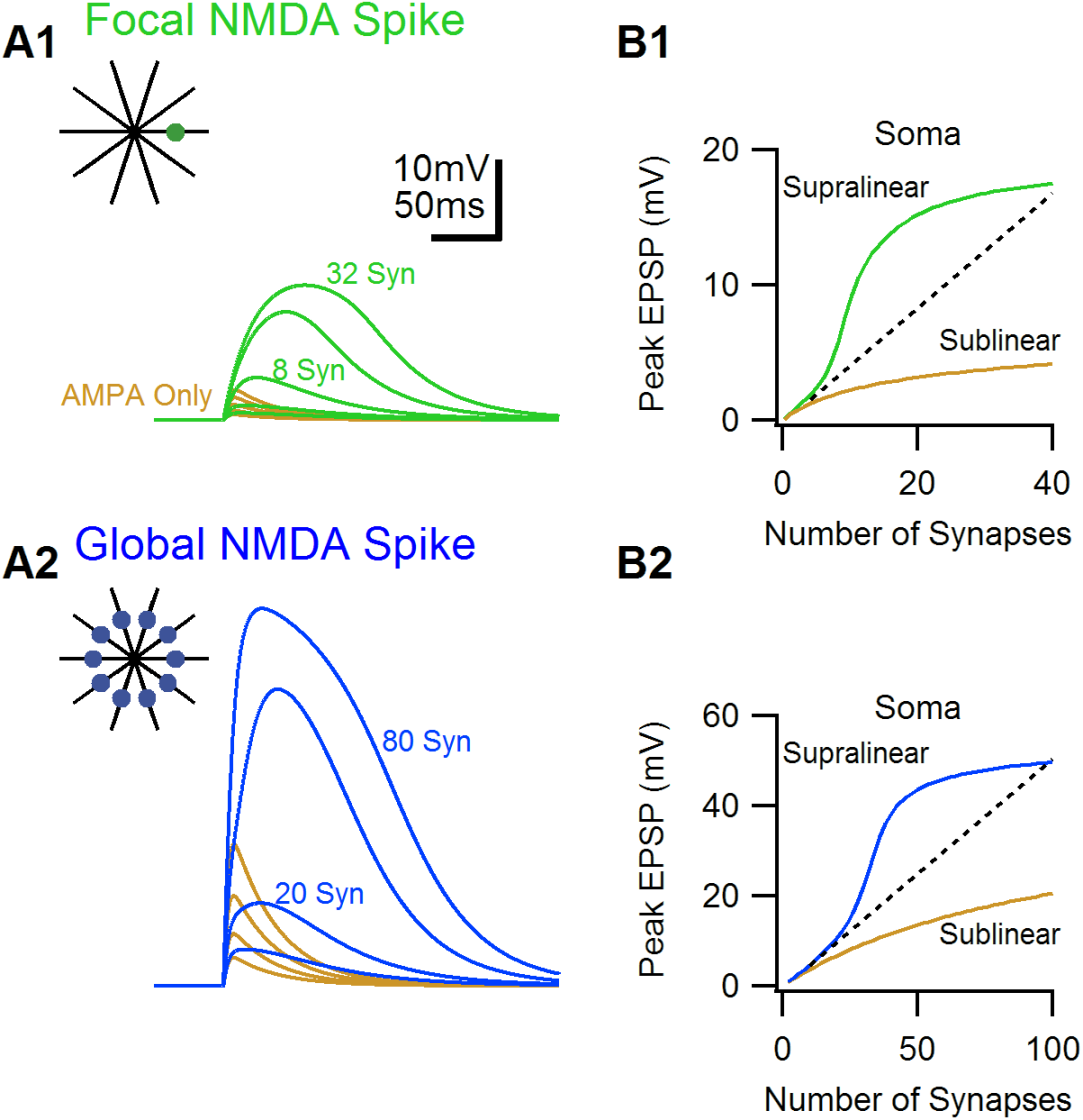
Examples of simulated focal and global NMDA-spikes. Simulated glutamatergic inputs were activated either on a single dendritic location (focal distribution, A1, marked by the green point), or on all the branches of the cell (global distribution, A2, blue points). A, Somatic voltage waveforms for different activation intensities (4, 8, 16 and 32 synapses) for the focal distribution (A1), and 10, 20, 40 and 80 synapses for the global distribution (A2). Synaptic activation of both distributions produced NMDA-spikes, as was evident by a large supralinear increase in the somatic amplitude. For comparison, AMPA-only EPSPs for similar intensities of activation are shown in brown.B, Peak somatic EPSP amplitude as a function of stimulation intensity for the two distributions. Dotted lines denote a linear extrapolation of a unitary EPSP. Color coding as in A.

To examine the factors that govern NMDA-spike initiation, I first used a simplified model of a passive neuron with ten symmetric dendrites connected directly to the soma. Glutamatergic synapses containing AMPA and NMDA receptors were the sole providers of synaptic inputs. Synaptic integration and dendritic spike generation were examined in two distribution scenarios; in the focal distribution all synapses were placed on a single dendrite. In the global distribution inputs were activated on all branches simultaneously (Fig 1).

Synaptic activation in both distributions yielded highly non-linear integration of the synaptic inputs (Fig 1). At low stimulation intensities, the somatically recorded EPSPs scaled linearly with synaptic number. As the number of active synapses increased, a strong supra-linear somatic response was obtained for both synaptic distributions, followed by a saturation of the somatic potentials at stronger stimulation (Fig 1). The supra-linear integration was dependent on NMDA receptors, as similar simulations with AMPA-only containing synapses caused only linear or sub-linear integration (Fig 1, brown).

Crucially, for the global distribution scenario, the supra linear integration was observed with just 3.7 active synapses per each branch, which is significantly less than the 8.5 synapses required to generate a an NMDA-spike with a focal synaptic placement (Fig 1B). This shows that, for the global distribution synaptic integration became dependent on crosstalk with inputs on other dendrites. Thus the global distribution produced a spike by summing of multiple branches, consistent with the mechanism of the global NMDA-spike [18].

### Similar spatial-temporal properties of NMDA-spike generation for focal and global synaptic distributions

**Fig 2:**
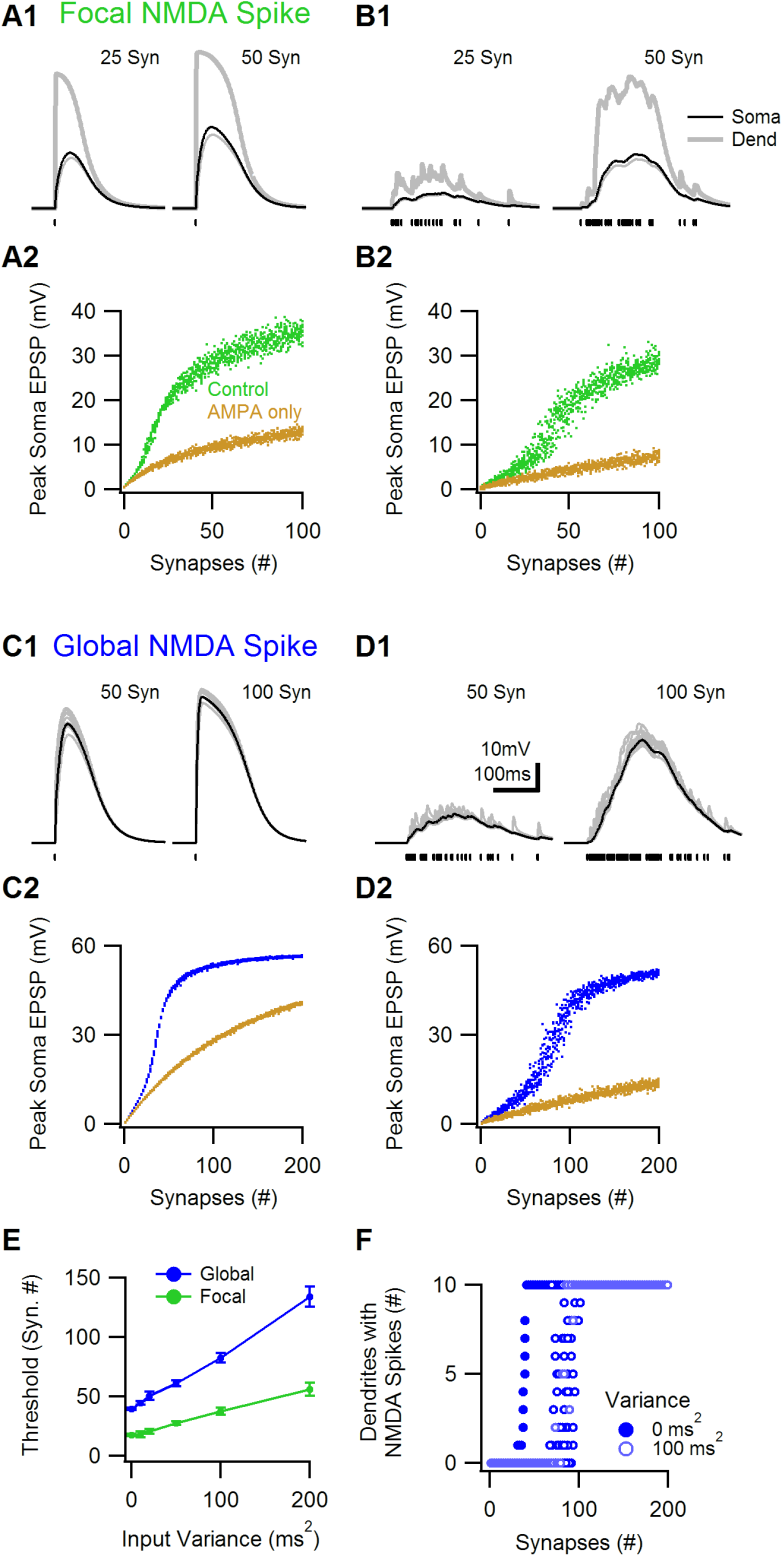
Spatial-temporal parameters of NMDA-spike generation. Simulations were performed in a similar morphology as in Fig 1. Simulated glutamatergic inputs were activated either on random locations on a single dendrite (focal distribution, A-B), or on all the branches of the cell (global distribution, C-D). A1, Somatic (black) and dendritic (grey, the branch where synaptic inputs were placed marked in bold) voltage waveforms for 25 (left) and 50 (right) simultaneously activated synaptic inputs. NMDA-spikes were elicited in both cases. Bottom, dots indicate the timing of synaptic input. Inset, schematic of the synaptic distribution on a single branch. A2, Similar simulation for asynchronous synaptic activation. An NMDA-spike was present only with 50 synaptic inputs. B1, The peak EPSP recorded at the soma for various spatial distributions of simultaneously activated synaptic inputs. NMDA-spike generation threshold was similar for various random distributions (green, brown, AMPA-only activation; compare to Fig 1, B1). B2, The number of inputs required to elicit NMDA-spikes was considerably elevated and more variable for asynchronous synaptic input. C and D, similar to A and B for synaptic inputs randomly distributed on all 10 dendrites. E, NMDA-spike threshold as a function of variance between the timing of synaptic inputs. F, The number of dendrites in which an NMDA-spike (measured as >30mV EPSP, lasting for more than 30ms) was present vs. the number of globally distributed synaptic inputs.

In a physiological activation, the synaptic input is not expected to be simultaneous, and not all synapses will be active at the same location. Previous work revealed a loose spatial-temporal requirements for focal NMDA-spike initiation, showing that excitatory inputs over the entire dendrites of a cortical pyramidal cell, active within 100-200ms, can combine together to promote a dendritic NMDA-spike [6, 9, 19]. Fig 2A and 2B shows that these properties of the focal NMDA-spike were readily replicated in the morphologically simple cell simulation. 8.3±1.2 synchronously activated inputs reliably triggered a focal NMDA-spike (Fig 2A1 and 2B1). Decreasing the temporal correlation between input activation increased the number of synapses needed to cross spike threshold to 28.5±6.6 for activation variance of 100 ms^2^ (Fig 1A2, 1B2 and 1E), in line with previous reports [19, 20].

Qualitatively similar results were found for the global synaptic distribution; 39.6±1.4 synchronous and 82±9.4 asynchronous (variance of 100 ms^2^) inputs could trigger the global NMDA-spike (Fig 2C–2E). The temporal variability of synaptic drive increased the number of inputs required to trigger an NMDA-spike by a similar ratio for global and focal synaptic distributions (Fig 2E). Interestingly, the spatial distribution of synaptic inputs did not affect the characteristics of the global NMDA-spike generation; for the simultaneous input, the somatic depolarization was similar for various permutations of synaptic placement (Fig 2D1 and 2E) In this condition the global NMDA-spike was almost an ‘all-or-none’ phenomenon, either absent from, or present on all dendrites (Fig 2F). Even when the temporal variance was very high, the number of active inputs predicted well the somatic depolarization level and NMDA-spike presence on the dendrites (Fig 2D2–2F). These results demonstrate very loose spatial-temporal requirements for the global NMDA-spike generation. Activation of ~100 glutamatergic synaptic inputs activated at random locations over the entire dendritic tree of the simulated cell, within a time window as wide as 200ms, could reliably lead to a global NMDA-spike generation (Fig 2F).

### The NMDA-spike is governed by the input resistance

Next I analyzed the factors that regulate NMDA-spike generation and compared the resulting predictions first in the simple compartmental model and then in actual morphologies of principal cortical cells.

#### Focal NMDA-spike(clustered synaptic distribution)

The focal NMDA-spike is initiated when synaptic NMDA conductance is strong enough to produce a large local EPSP that effectively removes Mg^++^ block from neighboring NMDARs and starts the regenerative feedback loop[5, 21].

Assuming an idealized passive cell and because the timescale of the NMDA-spike is much slower than the membrane time constant, we can disregard capacitive currents, the intensity of synaptic activation needed to reach a focal NMDA-spike threshold and to maintain an NMDA-spike are set by Ohm’s law

Isyn=VRIN

Where I_syn_ is the synaptic current, V is the voltage threshold for NMDA spike generation (typically ~20mV above the resting membrane potential) and R_IN_ is the local (dendritic) input resistance (R_Dend_).

Accordingly, dendritic locations with a higher R_Dend_ would require less synaptic activation to generate a focal NMDA-spike [9, 22]. Once initiated, the focal NMDA-spikes attenuate to other regions of the cell, including the soma. The attenuation factor is always smaller than, but can be quite close to R_Soma_/R_Dend_, where R_Soma_ is the somatic input resistance [23–28].

These considerations explain why distal dendrites (which typically have a higher R_Dend_) have the lowest threshold for focal NMDA-spikes and lowest peak somatic depolarization [9, 29].

#### Global NMDA-spike (distributed synaptic distribution)

What happens when synaptic inputs are activated on more than one branch? Electrical signals can flow across the cell, and therefore, synaptic inputs can influence each other even when they are not physically located on the same postsynaptic branch. To what extent this influence goes depends, among other facts, on the input and transfer resistances between each and every synaptic input and thus are too complex to be formulated precisely for a given neuronal morphology.

Fortunately, the outcome of a distributed synaptic integration can oftentimes be well approximated by a single indicator of the input resistance of the compartment where synaptic inputs were activated. In the simplified neuronal morphology presented above, the soma is the effective site of synaptic integration, even if no actual synaptic inputs are active directly on the cell body. In this cell, the soma can be thought of as the place where all NMDA receptor currents are summed up, the degree of the integrated postsynaptic depolarization is determined by the local electrical properties, and the resulting EPSP is sent back to the synaptic sites at the dendrites. Cells with higher somatic input resistance (R_Soma_) would in theory require less synaptic inputs to reach NMDA-spike threshold. Using Ohm’s law equations again, the number of synapses to generate a global NMDA-spike is inversely proportional to R_Soma_.

There are two important caveats to this analysis. First, the soma may not be the integration site of the synaptic inputs. For example, in some cortical pyramidal cells, inputs to the apical dendrites are integrated locally (see below). Furthermore, signal transfer between ‘sister’ branches, which share a parent dendrite, does not involve the soma. The second caveat is the possibility for a simultaneous local and global regenerative, NMDA receptor drive, synaptic integration.

In a complex synaptic activation, some glutamatergic inputs may be more clustered or isolated from the main integration site and participate in a focal form of an NMDA-spike, while other inputs integrate globally. As I will show next, local regenerative currents will reduce from the potential to form a global regenerative NMDA receptor response. Focal integration of synaptic inputs would be evident as smaller global NMDA-spike.

This theoretical analysis places a strong emphasis on the R_IN_ as the main determinant of NMDA-spike properties. In next sections I will test this prediction and examine how the morphology of a cell and changes R_IN_ affect the properties of the NMDA-spikes.

### Neural morphology has a strong influence on the rules of NMDA-spikes activation

It is possible to adjust the morphology of any neuron in a simulation so that to change only the somatic or the dendritic R_IN_. However, these manipulations would require fine tuning of multiple arbitrary parameters for each IR value. Besides being rather inelegant, these simulations would not provide intuition on how morphological variations between neurons can influence NMDA-spike initiation. Thus I opted to observe how four common morphological factors, which are the diameter and the length of the dendrites, the size of the soma and the number of dendrites affect NMDA-spike initiation. For each simulation I calculated dendritic and somatic input resistances and compared the expected and the actual NMDA-spike amplitudes and thresholds.

#### Dendritic diameter

**Fig 3:**
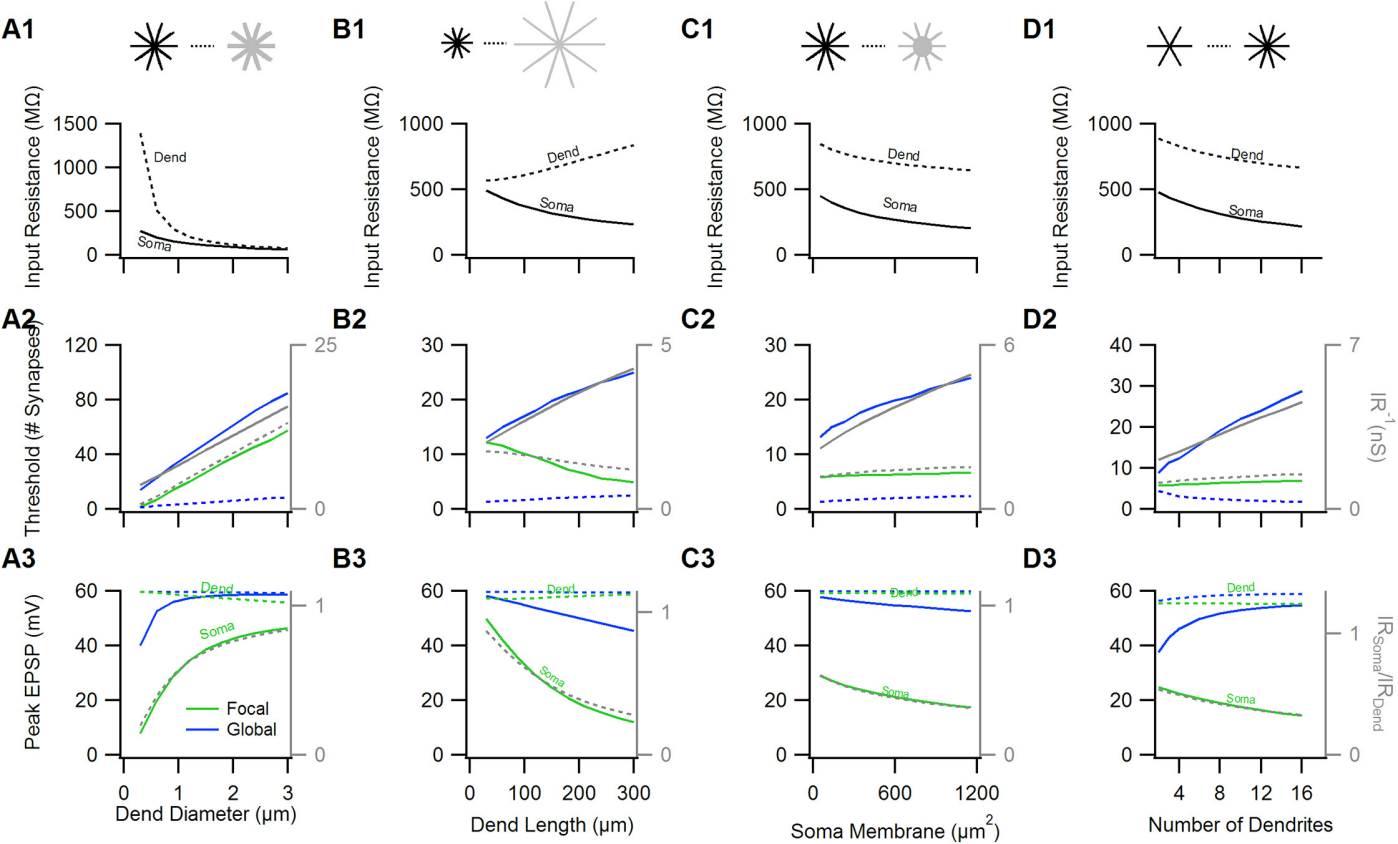
The impact of cellular morphology on the properties of the NMDA-spikes. The morphology of the artificial cell was varied as follows; in A The diameter of all dendrites was changed between 0.3 and 3μm, in B The length of the dendrites varied between 30 and 300μm, in C the membrane size of the soma scaled from 44 to 1170 μm^2^ and in D the number of dendrites increased from 6 to 16. For each manipulation, the following information is displayed: top, somatic (solid) and dendritic (dotted) R_IN_. Middle, synaptic threshold for focal (green) and global (blue) NMDA-spikes. For comparison, the dotted blue trace shows the number of synapses on a single branch that initiated a global NMDA-spike. Focal/global spike thresholds were well correlated with the inverse of the dendritic (dotted grey)/somatic (solid grey) R_IN_ values. Bottom, peak somatic (solid) and dendritic (dotted) spike amplitudes for the focal (green) and global (blue) NMDA-spikes. The somatic amplitude of the focal NMDA-spike was almost identical to the ratio between the somatic to the dendritic R_IN_ values (dotted grey).

Increasing the diameter of the dendrites in a neuron reduces both R_Soma_ and R_Dend_ (Fig 3A1).In agreement with the theoretical considerations, both the focal and the global NMDA-spike thresholds increased with dendritic diameter (Fig 3A2) and the peak somatic amplitude of the focal NMDA-spike scaled well with the R_Soma_/R_Dend_ (Fig 3A3). The peak somatic EPSP amplitude of the global spike was near the reversal potential of the NMDAR for diameters>1μm, but was reduced for narrower ones, due to especially high efficiency of focal NMDA-spike generation at the thin dendrites (Fig 3A2, green) and generation of a mixed focal-global NMDA-spike.

#### Dendritic length

Variations of dendritic length pose an especially interesting case of effect of morphology on NMDA-spike initiation parameters, as cells with longer dendrites have larger R_Dend_, but at the same time smaller R_Soma_ due to increased total cell surface (Fig 3B1). Accordingly, spike thresholds followed opposing trends. In neurons with very short branches, the focal and global NMDA-spikes had very similar thresholds and peak EPSP amplitudes (Fig 3B2). For morphologies with longer dendritic trees, the global NMDA-spike threshold increased as an inverse of R_Soma_, while the threshold and the amplitude of focal NMDA-spike decreased (Fig 3B2 and 3B3),in line with theory predictions.

#### The size of the soma

Changes in somatic size mainly impact the R_Soma_ and to a smaller degree the R_Dend_(Fig 3C1). As a consequence, the threshold of the focal NMDA-spike hardly changed for various somatic sizes, while the threshold for the global spike increases by about 40% with doubling of the somatic size (Fig 3C2).

#### Number of dendrites

Last I examined what is the effect of the number of dendrites on NMDA-spike initiation. The number of dendrites in a cell mainly affects R_Soma_, and has a small impact on the R_Dend_ (Fig 3D1). Accordingly, focal NMDA-spike threshold did not change significantly between morphologies with different number of dendrites (Fig 3D2, green). Having a small number of dendrites did significantly increase R_Soma_, and reduced the threshold for the global NMDA-spike (Fig 3D2, blue). Nonetheless, the threshold for the global spike on each branch actually increased (Fig 3D2, dotted line). This skewed the balance between focal and global NMDA-spike initiation towards more local integration in a cell with fewer dendrites and reduced global NMDA-spike amplitude (Fig 3D3, blue).

### Effects of morphology of reconstructed cortical neurons on the parameters of NMDA-spikes initiation and propagation

Simulations presented below examined whether the lessons learned from artificial neural morphologies can be applied to cortical neuron classes of principal (excitatory) neurons, where NMDA-spikes were demonstrated before[5–7, 10, 12, 18, 30]. For this simulation I used reconstructed neurons, and activated synaptic inputs either over the entire dendritic tree (global distribution) or on a randomly selected terminal dendrite (focal distribution).

Neural morphology of principal neurons varies widely across cortical layers. Layer 5b neurons have a large soma connected to far reaching basal and apical trees. Neurons in superficial layers (layer 2/3 and layer 4) are smaller, with less branches and as is the case for spiny stellate layer 4 neurons, with no apical tree. Based on predictions of the simulations presented so far, the difference in morphology between the deep and superficial layers neurons should affect the probability of focal vs. global NMDA-spike generation and synaptic integration. As I will show next, activation of synaptic inputs on the reconstructed cells produced responses which were in good agreement with simulations in the morphologically simplified cell.

**Fig 4:**
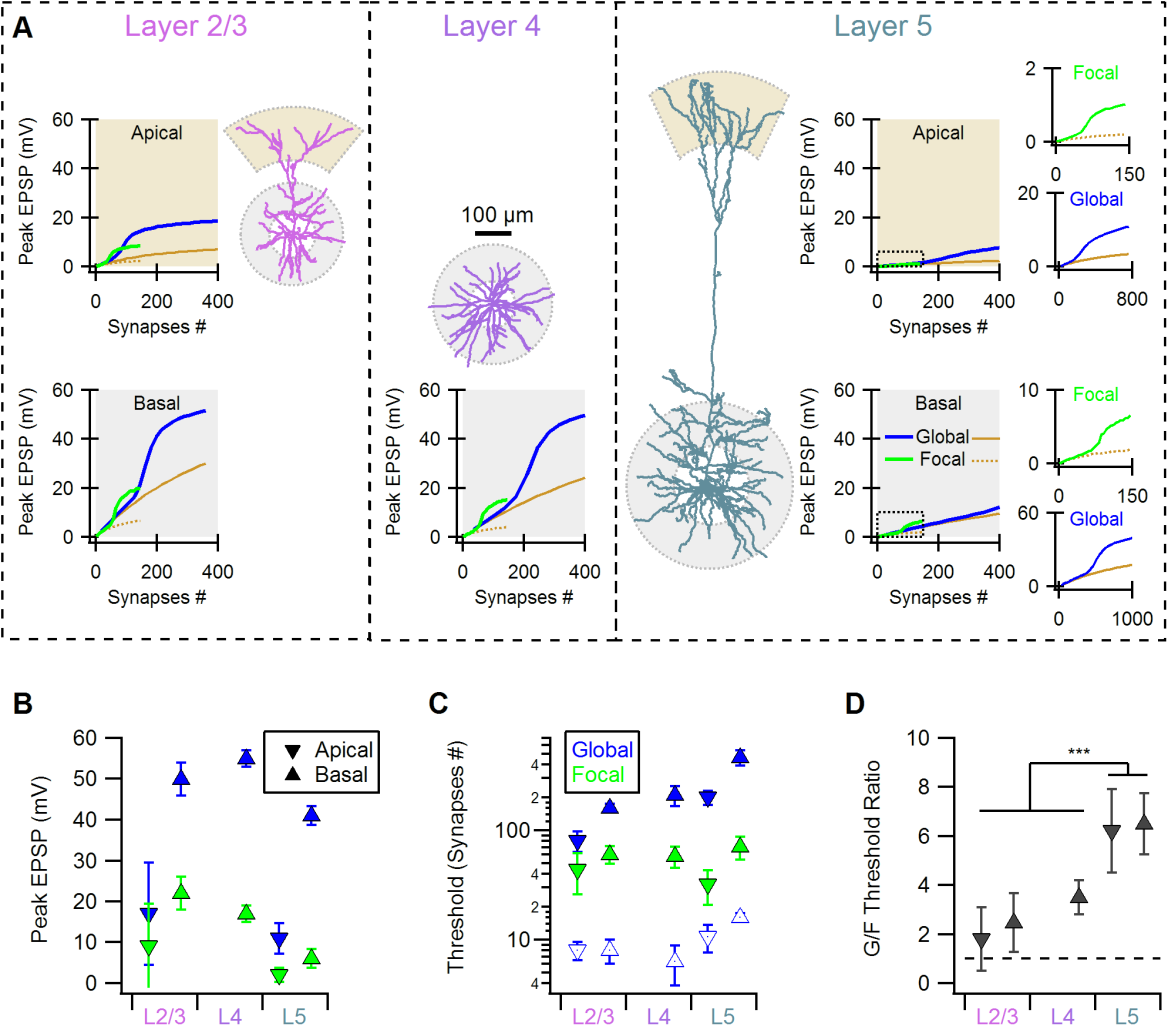
Morphological differences between principal cortical neurons predict distinct rules of NMDA-spike initiation. A, Examples of the morphologies and the input-output relations in simulated cortical layer 2/3 pyramidal (left), layer 4 spiny stellate (middle) and layer 5b pyramidal (right) neurons. For each cell class the plots show the peak somatic EPSPs evoked by a representative focal (green) or global (blue) synaptic distribution for basal and apical (where applicable) dendritic trees. For comparison, AMPA-only stimulation is shown in brown (solid for global and dotted for focal distributions). For layer 5b pyramidal neurons inserts show the full scale of the responses. B, Summary of the peak somatic NMDA-spike amplitude for the different dendritic trees (apical ▼ and basal ▲) in the three classes of cells for the focal (green) and the global (blue) synaptic distributions. C, The number of synaptic inputs (synaptic threshold) required to generate the NMDA-spikes, color coding as in B. Open symbols show the average number of synapses per each branch that lead to global NMDA-spike initiation. D, The ratio between the synaptic thresholds for global and focal NMDA-spikes is significantly different between superficial and deep cortical neurons (***p<0.001, ANOVA, Tukey’s HSD test)

As can be seen from Fig 4A, stimulation of all three classes of cells could generate focal and global NMDA-spikes, but with dramatically different rules for NMDA-spike generation and spike amplitude at the soma. As expected, the global NMDA-spikes were found to have a similar amplitude in all examined cells, when synaptic inputs were restricted to the basal trees (Fig 4A and 4B, blue). Global NMDA-spikes at the apical dendrites reached similar depolarization next to the main bifurcation, but were lower at the soma, due to EPSP attenuation along the apical trunk(Fig 4A and 4B, blue). Somatic focal NMDA-spike amplitudes was much lower in layer 5b pyramidal cells compared with layer 4 and layer 2/3 neurons (Fig 4A and 4B, green), as was indeed found experimentally [5, 7, 18].

In parallel with the considerable different morphologies across the cortical layers, the actual number of synaptic inputs that were required to generate global NMDA-spikes was strikingly different between deep and superficial layers. 160±13 / 210±44 randomly distributed inputs could trigger global NMDA-spike in the basal trees of layer2/3 / layer 4 cells, while 460±60 synapses were needed to produce a similar spike in layer 5bbasal dendrites (Fig 4A and 4C, blue, n = 3 morphologies for each cell type).The difference in apical dendrites was less pronounced, with 76±17 vs.205±29 synaptic inputs for layer 2/3 and layer 5b cells (Fig 4A and 4C, blue). The threshold for the focal NMDA-spike was more conserved between different cell types, with 60±11 and 43±17 synapses for the basal and apical trees of layer 2/3 neurons,58±13 synapses for layer 4, and 71±17 and 32±11 synapses for basal and apical dendrites of layer 5b cells (Fig 4A and 4C, green, n = 15 randomly selected terminal dendrites for each cellular morphology).In conclusion, in principal neurons in superficial layers, dendritic spike initiation requires relatively low number of synaptic inputs and leads to pronounced somatic depolarization. While in these cells the difference between focal and global NMDA-spikes thresholds was only two fold, in layer 5b cells, the number of synapses required for spike generation showed a clear preference (>5 fold) for focal NMDA-spike initiation (Fig 4D).

**Fig 5:**
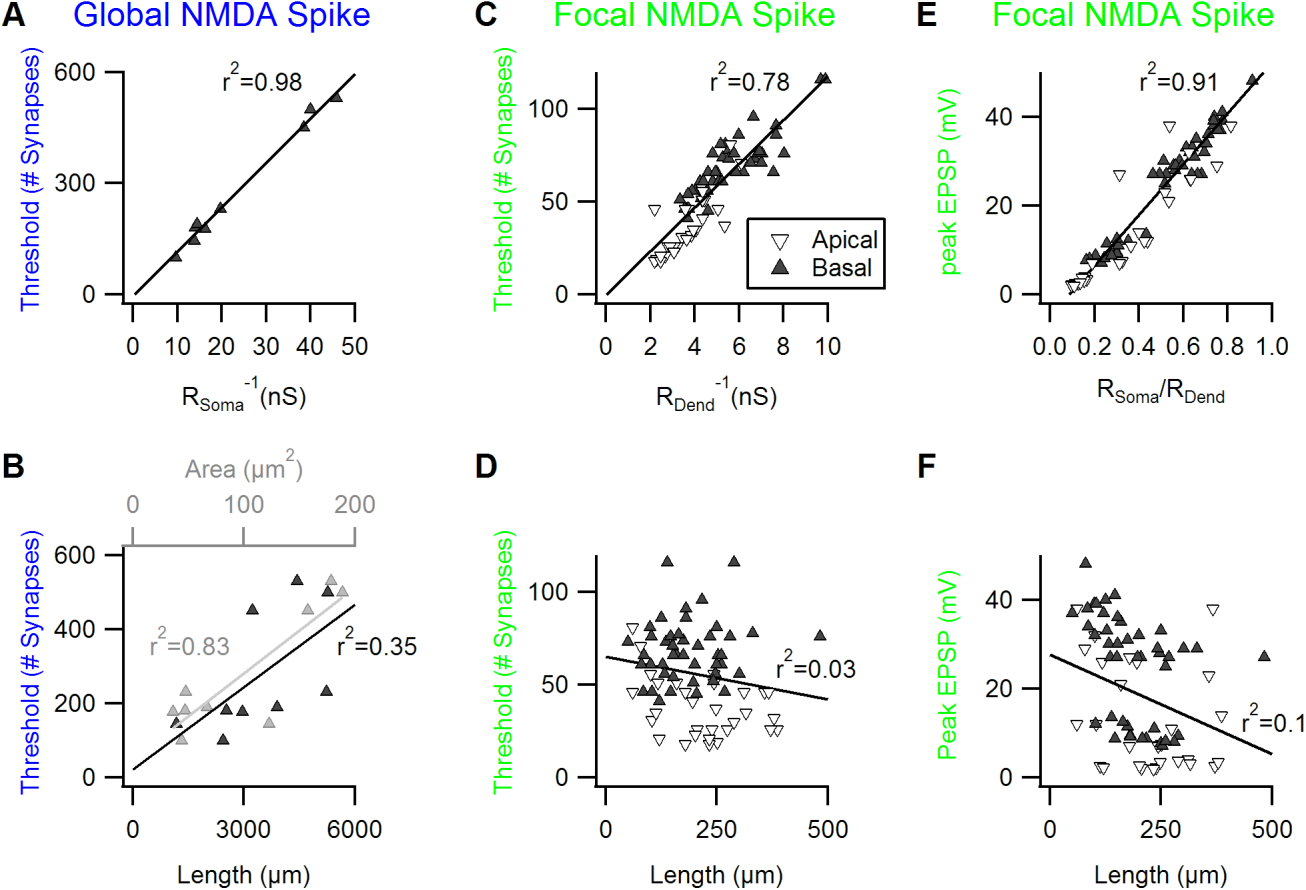
Dendritic and somatic Input Resistances determine NMDA-spike properties in realistic neuronal morphologies. A,B, The correlation between the number of synapses required for a global NMDA-spike generation in the basal dendrites and the inverse of somatic input resistance (A), soma size (B, grey) and the cumulative length of the dendritic trees (B, black) for the cortical cellular morphologies. Here and in the next plots the solid line indicates a linear fit to the data. C, D, A good correlation was found between the number of synapses required for focal NMDA-spike generation to the inverse of the focal dendritic input resistance(C) but not to the length of the stimulated branch (D), both in basal (black) and apical (grey) dendrites. E, F, A high correlation was found between somatic amplitude of the focal NMDA to the ratio between the somatic and dendritic input resistances (E) but not to the length of the stimulated branch.

As was the case for the simulation of a morphologically simplified cell, focal and global NMDA-spike thresholds followed the R_Dend_ and R_Soma_ respectively, but not somatic size or dendritic length (Fig 5A–5D). Likewise, the peak focal NMDA-spike amplitude correlated well with R_Soma_/R_Dend_ ratio but not with the length of the stimulated dendrite (Fig 5E and 5F).

### Differences in synaptic integration between cortical layers

Previous experimental and modeling work on layer 5 pyramidal neurons revealed a distinct synaptic integration scheme mediated by focal NMDA-spike generation[6, 15]. While inputs to the same dendrite summed supra-linearly, synaptic activation between branches was found to be linear, due to compartmentalization of focal NMDA-spike generation. The lack of inter-branch interaction is thought to enable neurons to have multiple independent integration units, each performing its own non-linear summation [13]. In theory, such scheme may enhance the computational abilities of a neuron [13, 16, 17, 31–33].

As I have shown here, all cortical neurons can in principle generate global NMDA-spikes. By definition, global spikes arise following cooperative activation of synaptic inputs on multiple dendrites, which is in odds with inter-branch independence. This finding raises two important questions; first, if synapses summate linearly when distributed on two branches (in line with previous findings), but strong interactions are present for stimulation of a large number of different dendrites, what is the limit of independent multi-unit integration(or in other words, how many dendrites can be active simultaneously and sum linearly at the soma)? Second, as focal and global NMDA-spikes have a less distinct synaptic generation thresholds on superficial layers neurons, does synaptic integration in these cells follows the one found for layer 5b cells?

To answer these questions I examined multi-branch integration in deep and superficial neural morphologies. In each cell I activated synapses on selected branches, measured the somatic response to activation of each individual dendrite, calculated the ‘expected’ linear summation of the responses and compared it to the ‘actual’ combined simultaneous activation of all synaptic inputs.

**Fig 6:**
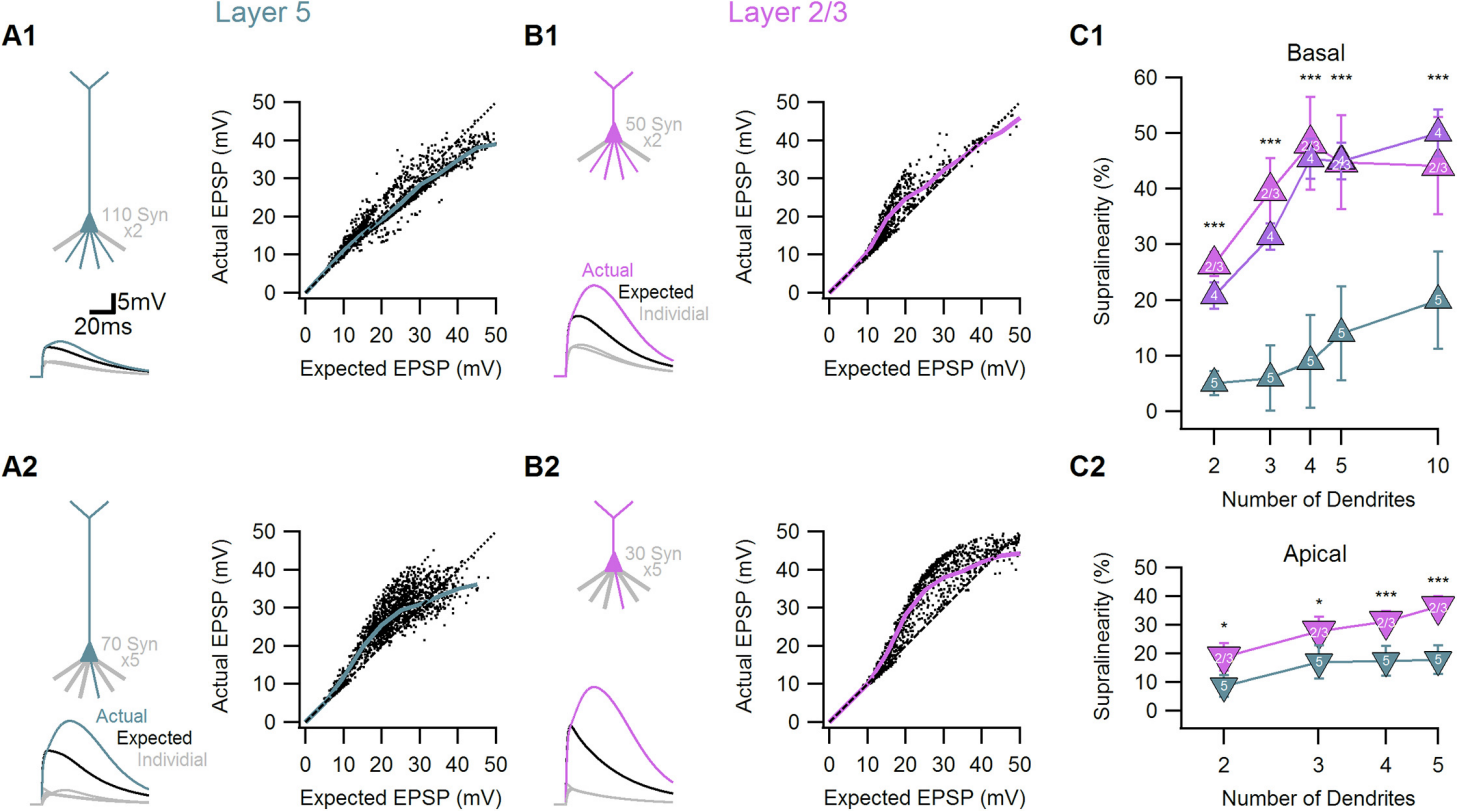
Subthreshold dendritic summation follows different rules in superficial and deep cortical layers neuronal morphologies. Synaptic summation was probed by comparing between the linear summation of somatic responses following input activation on individual dendrites (Expected) to the simultaneous activation of all synaptic inputs (Actual). A, Synaptic input summation between two dendrites was almost linear (A1), whereas five dendrites summed supralinearly (A2) in a simulated layer 5b pyramidal cell. Left, example of the somatic EPSP following activation of single dendrite (grey), the ‘Expected’ linear summation of individual dendrites (black) and the ‘Actual’ response (cyan). The cartoon on left shows the number of active branches and the number of synaptic inputs on each branch. Right, summary of the actual vs. expected EPSP amplitudes for 1000 trials (black).Bold trace, moving average. Dotted line, linear summation. B, same as A for cortical layer 2/3 pyramidal cell morphology. Actual (magenta) synaptic input summation was highly supralinear both between two (B1) and five (B2). C, The degree of supralinearity (measured as the ratio between the actual to the expected responses) for the three classes of cortical neurons for summation between different number of dendrites in basal (C1) and apical (C2) dendritic trees. *p<0.05,***p<0.001 between superficial and deep layers (ANOVA, Tukey’s HSD test).

Synaptic integration on two dendrites in basal tree of layer 5 neurons, the only region where experimental data exists[6], revealed very low levels of non-linear interactions, which were on average 9±2% above expected, and were limited to a narrow stimuli ranges (Fig 6A1 and 6C1).Increasing the number of stimulated dendrites elevated the degree of supralinearity up to 20±9% for 10 branches (Fig 6A2 and 6C1). Similar findings were found for stimuli directed at apical dendrites of layer 5b cells (Fig 6C2).

Dendritic independence was lower in neurons from superficial layers. Stimulation of two branches inlayer 2/3 / layer 4 cells, produced actual responses which were 26±3% / 21±2% larger than the expected linear sum of individual dendrites (Fig 6B1 and 6C1). Activation of more dendrites resulted in additional increase in supralinearity, both for the basal and the apical dendritic trees (Fig 6B2 and 6C).

### Potassium channels have a differential effect on focal and global NMDA-spikes and can affect the integration rules in cortical neurons

The simulations performed so far were conducted in passive cells, lacking any voltage gated channels. Given the significant distinction between synaptic integration in different neuronal morphologies, the questions of how NMDA-spikes are shaped by voltage gated conductances and whether the latter influence synaptic integration become especially prominent.

**Fig 7:**
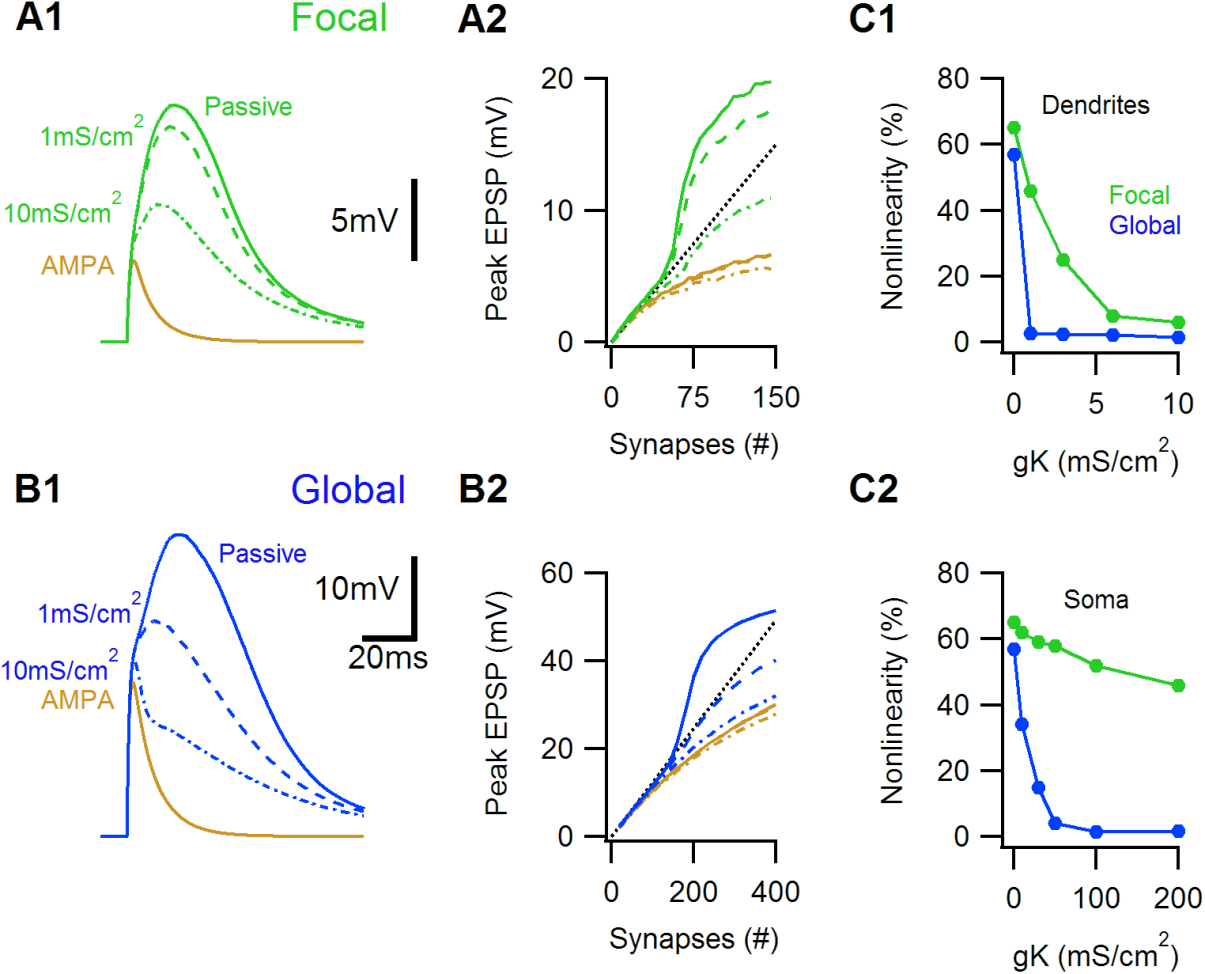
Voltage gated potassium channels have a differential effect on focal and global NMDA-spike amplitudes. A1, Somatic EPSP responses in a simulated layer 2/3 cell to 80 focally distributed synaptic inputs for a passive cell (solid), and in the presence of low or high dendritic potassium conductances (dotted). AMPA-only EPSP for the control case is shown in brown. A2, Peak somatic EPSP for the different potassium loads as a function of the number of synaptic inputs. Control, green; AMPA-only, brown. The dotted black line represents a linear extrapolation of a unitary EPSP. B, same as A for global distribution. C, Summary of the peak nonlinearity of the somatic EPSP as a function of the dendritic (C1) and somatic (C2) potassium conductances.

The role of potassium channels may be especially interesting, as they can limit the extent of supralinear integration by negating the excitability of NMDA-spikes[9, 15, 34, 35]. To measure this effect I first examined how activation of voltage gated potassium channels affects the focal and global NMDA-spikes. In the simulations presented here I used a generic delayed rectifying potassium conductance, similar results were found for A-type and M-type conductances. I found that irrespective of cellular morphology, potassium conductance reduced the amplitude of the global NMDA-spike noticeably more than that of the focal NMDA-spike, both when potassium channels were localized to the dendrites or to the soma only (Fig 7A). This is not entirely unexpected, given the significant pan-cellular depolarization evoked by the global NMDA-spike, which is expected to strongly activate voltage gated channels on many branches. The exact conductance and distribution of potassium channels is not known for many cells types, but is likely to fall within the range that has a large effect on the amplitude of the global NMDA-spike.

Potassium conductance reduced the magnitude of the global spike, but physiologically relevant potassium channel distribution could not prevent the effects of the global NMDA-spike on postsynaptic integration. Note that voltage gated potassium channels did not block the global NMDA-spike entirely; as can be seen from Fig 7A, the responses at the soma were considerably broader than AMPAR-only EPSP (blue vs. brown traces). Considering that the somatic depolarization in the case of the global NMDA-spike is well above threshold for the somatic action potential, it seems likely that this long lasting depolarization can trigger prolonged axosomatic firing.

In the last simulation I examined whether supra-linear between-branch summation found for the passive cells is evident from somatic firing pattern. In order to compare the expected to the actual firing rate, I first found the expected number of somatic action potentials to a linear summation of synaptic inputs. For that purpose I recorded somatic EPSC waveforms following activation of synapses on individual dendrites. Next I summed these individual somatic waveforms, and injected the resulting combined current back into the soma, and recorded the number of action potentials. This somatic firing to the expected EPSP was compared to the firing to actual combined activation of the synaptic inputs. In this way it was possible to determine whether the actual axosomatic spiking was supralinear (actual>expected firing rate), linear (actual = expected) or sublinear (actual<expected).

**Fig 8:**
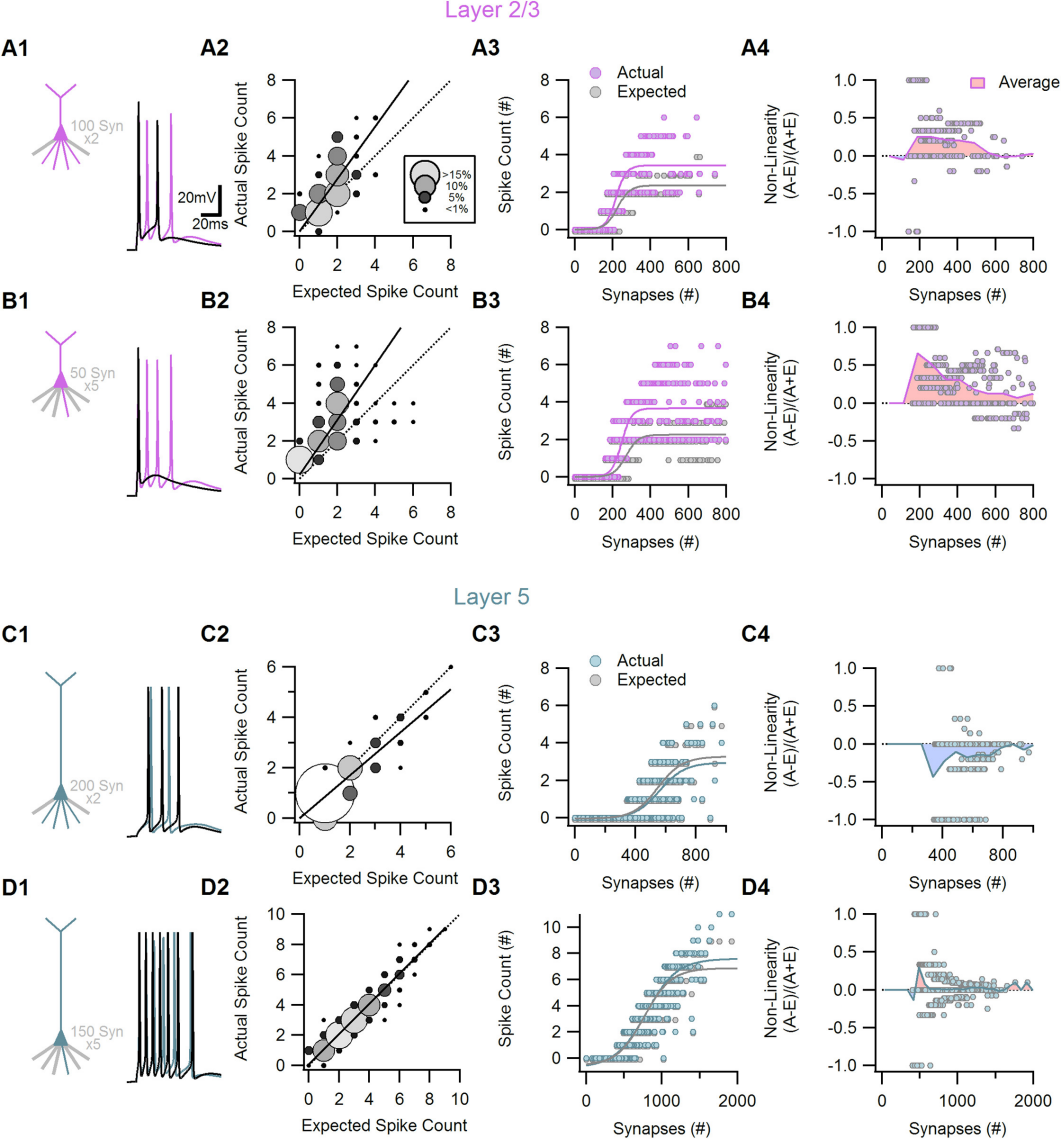
Simulated suprathreshold dendritic summation is supra-linear in superficial but not in deep layers cortical neurons. A and B, Axosomatic spike count resulting from dendritic summation between two (A) and five (B) dendrites was highly supralinear in a cortical layer 2/3 pyramidal neuron morphology. Linearity was tested by comparing between Actual synaptic activation to response to somatic injection of summed EPSPs from all active dendrites (Expected). A1, B1, cartoon of the number and distribution of synaptic inputs (left) and representative somatic potentials (right).Purple, actual simultaneous activation of all synapses. Black, expected somatic response to somatic current injection. A2, B2, The actual vs. expected AP counts for 3000 simulated suprathreshold summations. Circle size and color code for occurrence rate. Solid line, linear fit to the dataset, dotted line, linear summation. A3, B3, Axosomatic spike count as a function of the total number of activated synapses for the actual (purple) and expected (grey) summations. The data was fitted with sigmoid curves. A4, B4, The degree of non-linearity of the firing responses as a function of the number of activated inputs. Positive values indicate larger actual (A) responses that expected (E), zero is the linear case (actual = expected) and negative values for actual>expected cases. The dataset was analyzed with a moving average (purple curve). C and D, similar to A and B for cortical layer 5b pyramidal cell. The suprathreshold summation was remarkably linear between all branch number tested (2–10 branches).

Similar to the case of the subthreshold simulation, there were marked differences between the superficial layers and layer 5b cells; synaptic summation on layer 2/3 neuron often resulted in more APs then expected from the summated EPSPs of the individual branches (Fig 8A and 8B), primarily due to the temporary prolonged response of the cell to the synaptic stimulation [10, 20]. The supralinearity of the firing output was evident from the lowest suprathreshold stimulation intensities and persisted throughout a wide stimuli range (Fig 8, A3-4, B3-4). Layer 4 cells followed a similar trend (data not shown). In contrast, the firing in layer 5b cells was found to be linear and even slightly sublinear (reflected a saturation of the somatic action potential generation mechanism) over the entire suprathreshold regime, in line with independent between-branch synaptic integration units (Fig 8C and 8D). This was true for all synaptic distributions and stimulation intensities tested, indicating a genuine input-output transformation difference between different classes of cortical cells.

## Discussion

In this work I examined how synaptic signal integration with NMDA spikes is influenced by cellular morphology. I show a sharp distinction in sub- and supra-threshold synaptic integration rules between superficial (layer 2/3 and layer 4) and deep (layer 5) principal cortical neurons, mediated by two types of NMDA-spikes. The focal spike, which is generated by activation of clustered synaptic inputs, was extensively studied in previous studies [5–9, 11, 12, 18, 20, 36]. The global spike, described by [18], can be initiated by a more diffuse synaptic distribution. The two NMDA-spike modalities have very loose and comparable temporal requirements, but follow distinct spatial rules of synaptic integration. The global NMDA-spike depends mainly on somatic (when synapses are activated next to the cell body) input resistance, which set the threshold for the NMDA current that is needed for spike generation. This synaptic threshold is relatively insensitive to synaptic placement and thus conserved for various synaptic distributions. In contrast, the focal NMDA-spike is highly dependent on the dendritic environment. Small variations in branch morphology or distance of activation from the soma can have a profound effect on spike threshold and amplitude [9, 22, 29]. In passive cells, these spike parameters correlate to the reverse of input resistance and the ratio between somatic and dendritic input resistances respectively. If the site of focal NMDA-spike is shifted towards the cell body, the local input resistance decreases, which would require more synaptic inputs to initiate and boost the somatic impact of the spike [9, 29]. As the results of the simulations presented here clearly demonstrate, smaller neurons are generally more prone to NMDA spiking in general, with relatively small number of synaptic inputs required for the global form of the NMDA-spike[18]. This means that for a randomly distributed input, the cells in superficial cortical layers, which tend to have the smaller morphologies, are much more likely to generate a global NMDA-spike over a focal one. The situation is almost the opposite in layer 5b neurons, which have especially large soma and far reaching dendritic trees. According to the present analysis, these cells require activation of a very large number of synaptic contacts to generate the global NMDA-spike. Synaptic integration in such a cell could be plausibly carried out by focal NMDA-spikes, especially at the distal branches, where the spike threshold is lower[9]. From a computational perspective, one major difference between focal and global NMDA-spikes is the fact that the global spike is a singular phenomenon, while more than one focal spike can be present at any given time on distinct locations across the dendritic tree. This distinction leads to diverse possible computational roles for the two types of NMDA-spikes; the focal NMDA spike was suggested before to serve as a platform for dendritic parallel processing, synaptic clustering detector and possibly spatial-temporal encoder and decoder [20]. The global NMDA spike is much less sensitive to spatial synaptic distribution, thus it is likely to boost salient features of the synaptic drive, such as amplification of directional signals in directional selective cells [18].

The different integrative properties of superficial and deep cortical neurons found here may correlate to differential function of cells across the cortex. Cortical layers, despite having cells which are closely associated with each other, develop from different glial progenitors [37], are targeted by different sub- and intra-cortical regions which relay dissimilar information [38, 39] and have distinct molecular signatures [40], which may be perhaps enough to consider them to belong to separate ‘cortical strata’ [38]. This may not be so bizarre considering that recent studies in the avian brain found molecular and functional homologs to the mammalian cortex located in various disjoined parts of the forebrain [40].

### The effect of voltage gated channels in NMDA-spike assisted synaptic input integration

Previous studies examined integration of separate dendrites of large pyramidal cells and concluded that between branch interactions are sufficiently low to warrant them as independent [6, 13, 15, 41]. Voltage gated potassium channels are especially important in dampening excitability and as such can reduce nonlinear between-branch interactions, help to electrically isolate individual dendrites and perhaps boost independent multi-compartmental computations[13, 15, 34, 35]. Present work indicates that potassium conductance has a larger effect on the amplitude of the global NMDA-spike, apparently due to the presence of highly depolarized membrane potential over a wide area of the cell. Voltage gated potassium channels could completely linearize dendritic units’ summation in layer 5b cells (Fig 7). However, despite this marked effect on layer 5b pyramidal cells, potassium channels did not prevent supra-linear between branch synaptic integration and supra-linear somatic firing in superficial layers neurons, where summation between just two branches often generated somatic firing rate well above the expected one.

Obviously, somatic action potential or apical calcium spike initiation involve large voltage and conductance changes over a significant portion of the cell. However, NMDA-spikes are expected to be relatively insensitive to such signals[15]. Due to their amplitude, global NMDA-spikes are always expected to be followed by somatic firing, which may be especially prolonged due to the long plateau potential generated by the NMDA-spikes[10, 20]. This firing mode is well suited for stimulating dendritic units in downstream neurons, as NMDA receptors have a prolonged temporal integration window lasting tens up to hundreds of milliseconds[20]. The long integration period of the NMDA-spike means that a comparable number of inputs can generate spikes with lax temporal requirements. Previous and current estimates indicate that for the more ‘permissive’ superficial layers neurons, fewer than 200 synapses activated at random locations within a time window as wide as 200 ms can generate a global NMDA-spike[18].

### Evidence for NMDA-spike assisted cortical processing in vivo

It is important to note that the simulations presented in this paper were conducted for *in vitro* like conditions, with no background synaptic activity. In this way it was possible to isolate the passive effects of morphology. Background synaptic activation may profoundly change the rules of synaptic integration. For example, additional NMDA-rich glutamatergic inputs can directly increase the probability of NMDA-spikes initiation, while activation of AMPA receptors or inhibitory inputs can enhance or block the probability for spike generation due to their effects on the membrane potential and the input resistance[42, 43]. The spatial-temporal activation of inhibitory inputs is especially interesting, as inhibition was shown to affect NMDA-spikes differently depending on their location [44, 45]and the relative timing to NMDA-spike initiation[8, 46].

Cortical neurons in vivo experience high levels of network activity, both excitatory and inhibitory[47],.It is presently unclear whether and how NMDA-spikes affect synaptic integration *in vivo*. Several studies have found evidence for functional synaptic clustering [48–51], which might suggest a possibility for synaptic integration with focal NMDA-spikes (But see [52, 53]for counter evidence). Other studies found evidence for NMDA-spike generation [18, 30, 51, 54]or conversely no indication for a role of NMDA dependent regenerative processes [52, 53, 55].What evidence can be provided to demonstrate generation of a global NMDA-spike *in vivo*? As was observed in spiny stellate cells *in vivo* [18], the global NMDA-spike is characterized by a significant prolonged depolarization, most likely followed by somatic action potentials, with significant calcium influx. Clearly, these are not specific signals and may resemble multiple focal NMDA-spikes and calcium spikes. Focal NMDA-spikes can be differentiated from a global NMDA-spike with dendritic voltage recordings, which will show distinct active branches with focal, but not global NMDA-spikes. Calcium spikes often lead to homogeneous calcium influx, which may not be the case for the global NMDA-spike. The calcium signal of the global NMDA-spike may not correlate with the dendritic voltage, as dendrites may differ in the number of activated NMDA receptors [18].

Recently, calcium imaging and specific blockage of individual dendrites were used to probe the probability of NMDA-spike generation in layer 2/3 neurons [30]. Interestingly, hot spots of calcium entry to a single branch were as common as multi-branch spike like events, in which calcium, and presumably synaptic activity, was globally distributed. Although that study was not designed to differentiate between the possible mechanisms of the multi-branch spike, given the predicted predisposition of layer 2/3 pyramidal neurons for global NMDA-spike generation described in the present work, multi-branch calcium entry may in fact indicate generation of global NMDA-spike[18]. Hopefully, the findings of the present work will help to understand the black box which is the mechanism of synaptic integration in cortical cells.

## Materials and Methods

The simulations done in this work are available to download from the modelDB database [56].

Simulations were performed using NEURON 7.1 platform. The passive parameters of the cells used were membrane resistivity: 10,000 (Ωcm^2^), membrane capacitance: 1 (μF/cm^2^), axial resistivity: 100 (Ωcm).

The simplified neuronal morphology consisted of a soma (length and diameter of 20 μm; Volume of 1256 μm) and ten dendrites, each 0.5 μm in diameter and 150 μm in length. For simulations presented in Fig 2, only one of the listed morphological parameters was set as a free variable between simulations. Morphologies of cortical principal cells (layer 2/3 pyramidal, layer 4 spiny stellate, layer 5b pyramidal) were downloaded from the modelDB database and corrected for broken connections and unrealistic dendritic morphologies, most likely due to an automated reconstruction techniques used [56]. For each cell class 3 different reconstructed morphologies were tested.

To make the comparison between neurons insensitive to reconstruction methods and to compensate for the presence of spines, a diameter of 1 μm was assigned to all the fine dendrites. The calculated somatic input resistances from -0.1nA 100ms long current injections were 75±8 MΩ for layer 2/3 pyramidal neurons, 65±35 MΩ for layer 4 spiny stellate cells and 25±5 MΩ for layer 5b pyramidal neurons. These values correspond to the experimentally reported ranges for these classes in vitro [57–60].

In simulation with voltage gated channels, the basic channels types, fast sodium (500/0 mS/cm^2^) and delayed rectifier potassium (50/10mS/cm^2^) channels were inserted at the soma/dendrites for axosomatic action potential generation.

The integration time step of the simulation was 0.1ms. The simulations ran for 100ms.

Synaptic drive consisted of Alpha-Amino-3-hydroxy-5-methyl-4-isoxazolepropionic acid (AMPA) (0.3 nS) and NMDA (0.5 nS) receptors [18, 61, 62]. The kinetics of the synaptic conductances used were identical to [18];AMPA currents had an instantaneous rise time and a decay time of 2 ms. NMDA currents had a rise time of 2 ms and a decay time of 50 ms. The NMDA receptor conductance voltage dependence was modeled as follows: gNMDA = 1/(1+0.25·exp(-0.08·vm)) where vm is the local membrane potential.

The release probability was 1, all inputs were activated 20 ms from the beginning of the simulation. Dendritic spike threshold was defined as the number of synaptic inputs required to produce a 25% increase in somatic EPSP amplitude above a projected single synaptic input.

### Synaptic activation and analysis

For the simplified morphology presented on Fig 1 all synaptic inputs were placed at the center of the branch. For the focal synaptic distribution only one dendrite was innervated. For the global distribution, synaptic inputs were activated on all dendrites. For exploration of spatial-temporal rules of synaptic activation (Fig 2), synaptic inputs were randomly placed on either one (focal) or all (global) branches of the cell. For asynchronous inputs, the time of input activation was chosen at random from a distribution with a known variance.

For realistic neuronal morphologies focally distributed stimuli were placed on random locations on terminal dendrites, defined as the final 100μm-long regions from the branch endpoint, which are the sites of optimal focal NMDA-spike generation [9, 17]. For the global synaptic distribution, inputs were placed randomly on the terminal basal (directly connected to the soma) or apical tuft (above main bifurcation) dendrites.

For simulations of dendritic summation, synaptic inputs were placed on selected terminal dendrites. Synapses on each of the branches were activated sequentially, i.e. one at a time, the somatic EPSP recorded and summed to produce the ‘expected’ EPSP curve, which was then compared to the ‘actual’ response of the combined synaptic stimulation of all the branches. To generate an expected suprathreshold response synaptic inputs were activated sequentially on individual dendrites in somatic voltage clamp mode, the recorded EPSCs were linearly summed and injected at the soma in current clamp mode. There is a significant inter-branch variability between terminal dendrites in terms of their input resistance and distance from the soma. To compensate for this, I averaged the results of 1000 simulations for each summation condition. In each simulation run the individual branches were selected randomly.

The degree of nonlinearity for subthreshold inputs was computed as the ratio between the peak of the EPSP to the amplitude of the unitary EPSP multiplied by the number of synapses. For comparison between branch summation of nonlinearity was measured according to: (A-E)/E where A and E stand the actual and the expected peak EPSP amplitudes. As in many cases the number of APs generated by the cell was zero, a different metric was used to gauge the nonlinearity of suprathreshold responses, which was computed as (A-E)/(A+E), where A and E represent the number of APs for actual and expected stimulations respectively.
